# Supplementary material for: Pharmacophoric-constrained heterogeneous graph transformer model for molecular property prediction
Source: Commun Chem. 2023 Apr 3;6:60. doi: 10.1038/s42004-023-00857-x (PMC10070395; doi:10.1038/s42004-023-00857-x)
Supplement: Supplementary file 2 — Supplementary Information [file 42004_2023_857_MOESM2_ESM.pdf]

Supplemental Experimental Procedures

S1 Featurization Extraction

The feature extraction contains three parts: 1) Atom-level features. 2) Pharm-level features. 3) Junction-level features. We use RDKit to extract all features as the input of PharmHGT.

S1.1 Atom-level features

Table S1 and Table S2 show the atom and bond features we used in PharmHGT for atom-level view.

Features	Size	Description
Atom type	101	type of atom (e.g C,N,O)
Hybridization	6	sp, sp2, sp3, sp3d, sp3d2 or un-known
Number of H	1	number of bond hydrogen atoms
Degrees	1	number of neighbor atoms
Formal Charges	1	number of formal charge
Valences	1	number of valences
Gasteiger Charges	1	value of Marsilli-Gasteiger partial charges
Gasteiger HCharges	1	value of Marsilli-Gasteiger hydrogen partial charges
Aromaticity	1	whether this atom is part of an aromatic system
In ring	1	whether the atom is part of a ring

Table S1 Atom-level node features.

Features	Size	Description
Bond type	4	single, double, triple, aromatic
Stereo	6	none, any, E/Z or cis/trans
In ring	1	whether the bond is part of a ring
Conjugated	1	whether the bond is conjugated

Table S2 Atom-level edge features.

S1.2 Pharm-level features

Table S3 and Table S4 show the node and edge features we used in PharmHGT for pharm-level view.

Features	Size	Description
Pharmacophore type	8	type of pharmacophore (e.g NegIonizable,Acceptor, Donor)
TPSA	1	polar surface area
LogS	1	aqueous solubility
LogP	1	octanol/water partition coefficient
Number of C	1	number of carbon atoms
Number of H	1	number of hydrogen atoms
Number of O	1	number of oxdrogen atoms
Number of N	1	number of nitrogen atoms
Number of P	1	number of phosphorus atoms
Number of S	1	number of sulphur atoms
Number of F	1	number of fluorine atoms
Number of Cl	1	number of chlorine atoms
Number of Br	1	number of bromine atoms
Number of other atom types	1	number of other atoms types which are not mentioned above
Molecule weight	1	the weight of molecule
Have ring	1	whether there is a ring in pharmacophore
Number of ring	1	number of ring in pharmacophore

Table S3 Pharm-level node features.

Features	Size	Description
Reaction type	46	the reaction type between fragments based on BRICS rule

Table S4 Pharm-level edge features.

S1.3 Junction-level features

The junction-level node features is consisted of atom-level and pharm-level node features, see the Table S5.

Features	Size	Description
Junction information	1	the adjacency relationship between atoms and pharmacophore

Table S5 Junction-level information features.

Algorithm 1 algorithm caption

Input:
Graph $G = \{V, E\}$;
view types $t \in \mathcal{T}$;
Node features X_{v^t}, where $v^t \in V^t$;
Edge features $X_{e^t_{v,u}}$, where $e^t_{v,u} \in E^t$.

Ensure:
Neighbors find function $\Theta_{\mathcal{N}}$.
Readout attention function $\Theta_{\mathcal{R}}$.
Index inverse function $\Theta_{\mathcal{I}}$.

for t **in** $\mathcal{T}$ **do**
  $\mathcal{H}^t_0(E^t) \leftarrow X_{E^t}$, $\mathcal{H}^t_0(V^t) \leftarrow X_{V^t}$
  for $k = 1$ **to** K **do**
    $\mathcal{M}^t_k(V^t) \leftarrow \mathcal{H}^t_{k-1}(E^t_{\Theta_{\mathcal{N}}(V^t)})$
    $\mathcal{H}^t_k(V^t) \leftarrow \text{Linear}(\text{Cat}(\mathcal{H}^t_{k-1}(V^t), \mathcal{M}^t_k(V^t)))$
    $\mathcal{M}^t_k(E^t) \leftarrow \mathcal{H}^t_k(V^t) - \mathcal{H}^t_{k-1}(E^t_{\Theta_{\mathcal{I}}(V^t)})$
    $\mathcal{H}^t_k(E^t) \leftarrow \sigma(\mathcal{H}^t_0(E^t) + \mathcal{M}^t_k(E^t))$
  end for
end for
$\mathcal{H}(V) \leftarrow \text{Linear}(\text{Cat}(\Theta_{\mathcal{R}}(\mathcal{H}^{\mathcal{T}}_K(V^{\mathcal{T}}), \Theta_{\mathcal{R}}(\mathcal{M}^{\mathcal{T}}_K(V^{\mathcal{T}})))$
return $\mathcal{Z} \leftarrow \text{Readout}(\mathcal{H}(V))$

S1.4 Implementation and Experiments details

S1.4.1 Model iterations pseudo code

In the part, we given the PharmHGT model iterations pseudo code, see the algorithm 1.

S1.4.2 ROC curves of PharmHGT on classification tasks

The Fig.S1 is the ROC curves across 6 molecular properties prediction classification datasets, and Fig.S2 shown the performence of our model on each molecular property prediction classification dataset.

S1.4.3 Computing resources details

The Table S6 show the overall Computing resources performance comparison to the state-of-the-art methods on ESOL datasets. All models are trained or finetuned by one A100.

S1.4.4 More comparative experiments

We have added more experiments with the mentioned two datasets in our works. The Graphormer model is a 3D model which requires the 3D conformation information of small molecules. However, the data set for the molecular property prediction task does not provide 3D conformation. In order for the

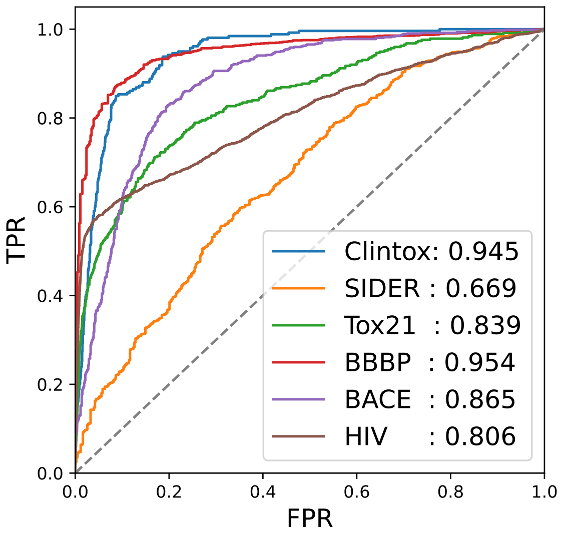

Fig. S1 Overall ROC curves on molecular property prediction classification tasks.

model to perform normal training and prediction, we use rdkit to get 3D conformation of each data set for training and testing with a random force field, and the final result is illustrated in Table S7 and Table S8.

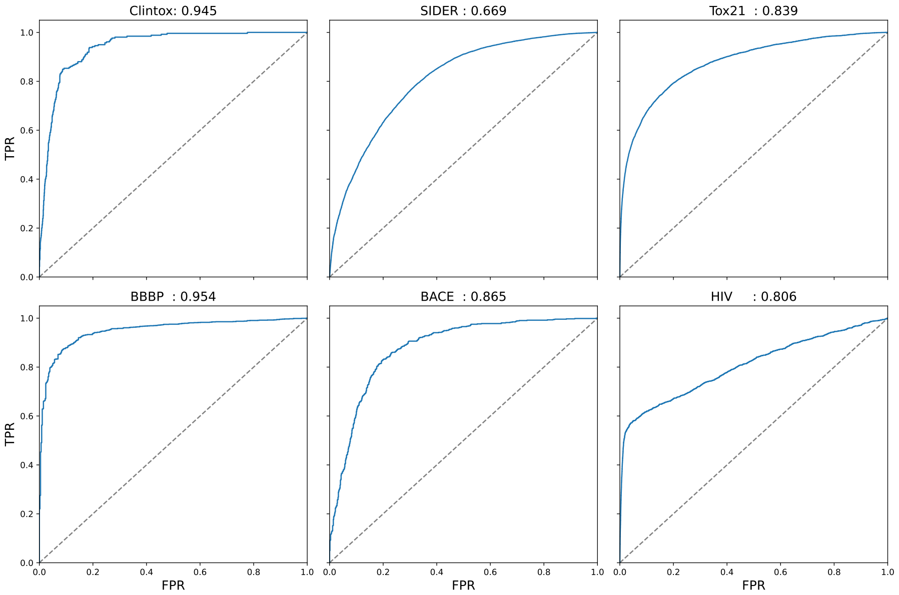

Fig. S2 ROC curves on each molecular property prediction classification tasks.

Table S6 Overall Computing resources performance comparison to the state-of-the-art methods on ESOL datasets. The results of baselines are obtained by us using a 5-fold cross validation with scaffold split and doing experiment on each task for one time.

Models	Params	Pretrain	Trainng time(s/epoch)	Training cost(s/fold)
AttentiveFP	0.65M	No	0.41	20.5
FragGAT	1.75M	No	0.72	36.0
MGSSL	2.72M	Yes	1.25	62.5
MPNN	1.06M	No	0.60	30.0
DMPNN	1.62M	No	0.80	40.0
CMPNN	2.00M	No	0.83	25.0
CoMPT	2.60M	No	1.80	72.0
GROVER_{base}	48.0M	Yes	4.79	115
GROVER_{large}	107M	Yes	5.83	140
PharmHGT	2.50M	No	1.20	24.0

6 Supplemental Experimental Procedures

Table S7 Overall Performance comparison to the state-of-the-art methods on molecular property prediction classification tasks. The results of baselines are obtained by us using a 5-fold cross validation with scaffold split and doing experiment on each task for one time. The values in this table are the Mean and standard deviation of ROC-AUC values.

Classification(ROC-AUC%, higher is better↑)						
Dataset	BBBP	BACE	ClinTox	Tox21	SIDER	HIV
Molecules	2039	1513	1478	7831	1427	41127
Task	1	1	2	12	27	1
Splitting strategy	Scaffold	Scaffold	Scaffold	Scaffold	Scaffold	Scaffold
Graphormer	93.6(2.41)	83.3(1.16)	88.1(3.80)	80.8(2.00)	62.0(1.20)	78.9(0.91)
PharmHGT	**95.4(1.15)**	**86.5(2.21)**	**94.5(0.42)**	**83.9(0.56)**	**66.9(1.63)**	**80.6(0.21)**

Table S8 Overall Performance comparison to the state-of-the-art methods on molecular property prediction regression tasks. The results of baselines are obtained by us using a 5-fold cross validation with scaffold split or Random split and doing experiment on each task for one time. The values in this table are the Mean and standard deviation of RMSE values.

Regression(RMSE, lower is better↓)						
Dataset	ESOL	FreeSolv	Lipophilicity	ESOL	FreeSolv	Lipophilicity
Molecules	1128	642	4200	1128	642	4200
Tasks	1	1	1	1	1	1
Splitting strategy	Random	Random	Random	Scaffold	Scaffold	Scaffold
Graphormer	0.858(0.032)	1.652(0.210)	0.839(0.020)	0.931(0.042)	2.019(0.752)	1.097(0.389)
PharmHGT	**0.680(0.137)**	**1.266(0.239)**	**0.583(0.026)**	**0.839(0.049)**	**1.689(0.516)**	**0.638(0.040)**
